# The History of Clinical Psychology in Greece: A Brief Review – Legal Deficiencies, Practical Dimensions and Challenges for the Future

**DOI:** 10.32872/cpe.12515

**Published:** 2024-12-20

**Authors:** Katerina Flora

**Affiliations:** 1 Department of Psychology, University of Western Macedonia, Florina, Greece; University of Vienna, Vienna, Austria

**Keywords:** clinical psychology, history, applied psychology, Greece, review

## Abstract

**Background:**

The history of clinical psychology in Greece spans more than 150 years. However, this branch of psychology concerned with the assessment and treatment of mental illness and psychological problems has not yet acquired the institutional and general recognition to which it is entitled.

**Aims:**

This article intends to highlight, chronologically, the basic elements of the history of clinical psychology in Greece, beginning with the important contribution of the work of philologist Panagiota Kazolea-Tavoularis.

**Results:**

From the first references in the context of medical studies during the 19th century, clinical psychology gradually develops through its application in pedagogical, laboratory, and clinical contexts to become an independent discipline alongside the consolidation of general psychology. Special mention is made of the scientists who pioneered this direction.

**Conclusion:**

The present review highlights historical milestones and concludes with the current situation, in which important steps have been taken. However, significant changes are needed at the institutional level.

## Highlights

- During the 19th century elements of clinical psychology were formulated in the context of medical studies.
- During the 20th century, clinical psychology developed more widely in pedagogical, laboratory and clinical contexts.
- The formation of clinical psychology as an independent branch took place in parallel with the consolidation of general psychology.

The purpose of this overview is to coherently present the history of clinical psychology, the branch of psychology concerned with the assessment and treatment of mental illness and psychological problems, in Greece. The basic bibliographic source for the writing of this article is the doctoral thesis of Panagiota Kazolea-Tavoularis (2001), titled “The history of psychology in Greece (1830–1987)”. This particular thesis gathers rich and original material. What is described in this article is, by a significant part, an extraction from the thesis, specifically related to clinical psychology. It is worth noting that there are no specific sources for the history of clinical psychology in Greece. Therefore, the source material mainly concerned the history of psychology in Greece in general, the history of applied psychology, and the history of psychiatry. Processing of the found material resulted in five general origins and strands from which clinical psychology in Greece is fed: medicine, especially neurology and psychiatry; philosophy and religion; educational science and pedagogy; psychoanalysis and psychotherapy; and the development of American and European (experimental and applied) psychology in general, with its various fields and disciplines.

This article follows a chronological order and concludes with a description of the current state of clinical psychology in Greece. The chronological order follows the work of Kazolea-Tavoularis’ thesis, with the addition of older and modern sources added to the basic elements included in the historical review.

## The Beginning – 19th Century

The development of clinical psychology in the 19th century was influenced mainly by medicine, particularly neurology and psychiatry, and its institutions (clinics, universities, and practitioners). Since the 19th century, applied psychology subjects have been taught in medical departments throughout Greece. Psychological theories concerning mental disorders and shaping the early practice of clinical psychology appeared during the 19th century and were taught at medical schools in the context of general pathology, since psychiatry as a specialty was unestablished. The influence of psychological factors on physical health was emphasized, which equated with the harmony of body and soul. Outside of a strict academic context, psychological issues were also referred to by doctors, and an early use of the term “psychosis” was recorded with reference to the history of Western psychiatry regarding the classification and treatment of phrenosis (Kazolea-Tavoularis, 2001). As early as 1885, scientific publications such as the *Journal Medical* had been established, in which doctors at the time – such as Ioannis Foustanos, a student of Jean-Martin Charcot – referred to hypnosis, removing any metaphysical dimension from the behaviour of the hypnotized.

Apart from academic, theoretical psychology, an applied clinical psychology was practised in psychiatric institutions, and it may have been influenced, to some extent, by religious and philosophical currents. According to Dimitris Ploubidis (1995), practical psychiatry was practised in several monasteries through wishes, exorcisms, and incantations but also with more violent means such as binding with chains and confinement. These practices were succeeded from 1838 on by the establishment of the insane asylum of Corfu and the Dromokaiteio sanatorium of Attica according to the standards of Western European sanatoriums based on the theories of Jean-Etienne Dominique Esquirol (Karamanolakis, 1998). These institutions were followed at the beginning of the 20th century by the Aegineteio (Athens) 1905, the public psychiatric hospital of Athens in the 1920s, and the public psychiatric hospitals of Thessaloniki and Souda, Chania (Crete) (Chartokollis, 1991).

During that period, psychological theories and clinical practices that the Greek doctors had learned from their studies in Europe were applied in these institutions, following the French school of degeneration of Valentin Magnan or the German school of psychiatry, which, at the end of the century, ended up in the Kraepelin classification of primitive dementia. There is a discrepancy in the diagnoses, as could be seen from the archives of the Corfu psychiatric hospital (1880–1885) where diagnostic terms such as lysomania, bimorphous phrenitis, bimorphous paranoia, rationalized paranoia, isodemia, enteric frenzy, and exphylogenic frenzy were mentioned. It is worth mentioning the work of two Greeks with international recognition, Grigorios Rosolimos in Russia and Konstantinos Oikonomou in Austria, in the field of neuropsychology during the same period.

In Greece in the 19th century, scientific events unfolded with a delay, as the state structures and educational ties were absent in the newly established state. Therefore, beyond the above references, psychology remained largely philosophical and was slow to evolve into an applied field.

## Clinical Psychology in the 20th Century

### Overview and Lines of Development

The 20^th^ century saw a more intensive and heterogeneous development of psychology and clinical psychology in particular. The development of clinical psychology in Greece came in two phases, the first lasting until about 1960 and the second following thereafter, as the discipline was largely established and consolidated as an independent scientific and practical field.

### The First Phase: Structure and Development

The first phase of the development of clinical psychology was strongly influenced not only by European and American psychology but also by pedagogy and psychotherapy. Institutes and professorships of psychology were established in Greece’s large cities and in institutions of applied clinical psychology in the fields of health and education.

In 1964, the first chairs of psychology were established at the universities of Ioannina and Thessaloniki (Kazolea-Tavoularis, 2001). Hypnosis and psychoanalysis were being practised elsewhere in Europe, and Greece imported knowledge about hypnotism for medical or parapsychological use. This sparked a dialogue that contributed to the awakening of interest in the soul as an entity and, by extension, in psychic phenomena. References to hypnosis, dreams, and parapsychological phenomena were noted in general psychology throughout the first half of the 20^th^ century. Some interested parties, however, leaned more towards mysticism, promoting religious beliefs and accusing scientific and research sources of being atheistic or materialistic.

#### Scientific Psychology in Universities and Laboratories

While psychology was taught as a general subject at the Philosophical School of Athens until 1908, as psychopathology at the Medical School of Athens, and as general psychology in teaching and secondary education, it was still part of philosophy courses at the beginning of the 20th century. The first psychology laboratory, which provided important services to student practice and research, was founded in 1926 at the University of Athens by Theophilos Boreas. The second such laboratory was established in 1935 in Thessaloniki, where the activities of Professor Georgios Sakellariou were particularly noteworthy; he founded the magazine *Prometheus* in 1951 and the Hellenic Psychological Society in 1955. At the same time, psychopedagogical research was being conducted by Alexandros Delmouzos, a professor of pedagogy in Thessaloniki and a student of Wilhelm Wundt.

The psychological laboratory of Athens was divided during the 1950s into four departments: psychological research, professional guidance, clinical psychology, and enlightenment of parents and young people; this was the first reference to clinical psychology at an academic level. The aims of the laboratory also included applied psychology. The work of S. Paraskeva-Sakka, who studied philosophy in Thessaloniki and psychology in the United States and was a colleague of Sakellariou, was noteworthy. Paraskeva-Sakka specialized in vocational guidance and psychoanalytic psychotherapy and translated and used many of the psychological tests still in use today.

#### Applied Psychology, the Beginnings

Psychological scientists were sought after by the Greek Army early in the 20^th^ century to help select and train conscripts in the most efficient use of new weapons and machines. In 1917–1918, a special committee drafted tests for the selection of aviators while selection methods were being implemented for conscripts and candidates for military schools. In 1948–1949, Sakellariou trained Army officers in the psychological laboratory at which intelligence tests were administered. Since the 1950s, the Army has used intelligence scales such as the Terman–Sakellariou scale and the Nikolaos Exarchopoulos Progressive Matrix Test scale. Additionally, applied psychology was used in the Greek Army until 1997, when the institution of psychosocial care was established to address conscripts’ problems.

Important contributions to the spread of psychology in Greece were made by two female pioneers, Sofia Gedeon and Aikaterini Striftou-Kriaras. They were collaborators of Nikolaos Exarchopoulos, the chair of pedagogy in the Laboratory of Experimental Pedagogy, who was succeeded by Spyridon Kalliafas. Kalliafas turned to the study of psychological issues, as can be seen by his publication of *Characters or Psychological Types* in 1935, in which he referred to older characterizations starting with Plato and Aristotle and continued with the psychoanalytic schools of Freud, Adler, and Jung. His study mentioned the relevant psychology of individual differences of Fechner, Charcot, Taine, Binet, and Stern and the contributions made by two Greeks, Nikolaos Exarchopoulos and Georgios Sakellariou. Kalliafas glorified Jung’s typology and was perhaps the most basic and important exponent of his work in Greece.

Konstantinos Specieris succeeded Kalliafas in 1953. In his work, *The Psychosynthesis of Man*, Specieris attempted a philosophical approach to the psyche; however, he emphasized the therapeutic effects of the psychoanalysis of Freud, Adler, and Jung. For the evaluation of the personality, he proposed his own psychograph type with 13 questions. Specieris’ work, *The Mental Life of Man* (Specieris, 1960) was a revision of his previous work based on the latest scientific findings. It referred equally critically to behaviourism, psychoanalysis, individual psychology, and the philosophy of existence. He also directly questioned the method of psychological tests and, in general, quantitative measurements that provide only partial knowledge of phenomena. He argued that the totality of mental life was greater than the sum of its parts in accordance with morphological psychology, while insightful understanding and deepening introspection were required to understand the human psyche.

Applied psychology was introduced in Greece at the beginning of the 20th century with the particularities that characterized Greek scientific and social reality at that time. Psychology was not clearly accepted as an independent scientific field, so discussion about Freud’s theories of psychology and psychoanalysis took place within the circles of pedagogy. The first translations of Freud’s works were published in the early 1900s, while at the same time the psychoanalytic movement was remembered mainly for its pedagogical application. Beyond Freud, Adlerian principles of pedagogy were taught in selected schools of the country and also in special education through the work of the pioneering pedagogue Roza Imbrioti.

The medical community in Greece initially had a negative attitude towards psychoanalysis, which favoured the demedicalization of psychoanalysis in Greece during the first period of its introduction. However, Dimitrios Kouretas, a neurologist and psychiatrist, delivered the first lecture on psychoanalysis in Greece in 1927. He contributed to the creation of the first psychoanalytic nucleus in Greece in 1947 with Andreas Empirikos, a poet and exponent of surrealism in Greece, and Georgios Zavicsianos, a psychiatrist, under the supervision of Princess Marie Bonaparte, a student and translator of Freud and president of the Paris Psychoanalytic Society. The name Nikolaos Drakoulides was also mentioned; he wrote a number of studies, including one on Freud and psychoanalysis (1936) and another on the psychoanalytic interpretation of art (1948). Psychoanalysis, however, was practiced clinically and was referred to as the new psychotherapeutic method with encouraging results since the 1930s. Prominent practitioners were psychiatrist Mihail Vlastos, psychiatrist Fotis Skouras, and neurologist–psychiatrist Konstantinos D. Konstantinidis, a professor at the Medical School of Athens and director of the Public Psychiatry of Athens. Countering this group was Georgios Zouraris, a member of the Institute for Sexual Research in Berlin, who favoured psychobiology and criticized concepts of psychoanalysis such as childhood sexuality and the Oedipus complex.

Given the interaction and mutual borrowing between disciplines and the theoretical issues of psychology and psychiatry (Tzavaras, 1991), a reference to psychiatry in Greece also concerned the history of psychology in the country, insofar as the conceptions of the “soul”, its functions, and its pathology were common points of concern for both. The popularity of psychological theories and therapeutic practices such as psychoanalysis and others, since it was only in 1963 that neurology and psychiatry were institutionally separated, erased the special characteristics of clinical psychology in institutions and as taught by university departments. After all, the concept of mental illness was part of a specific institutional framework, which, reflected the socio-economic conditions at the time as well as the ideological parameters in Greece, as Kazolea-Tavoularis (2001) commented.

#### Mental Health Services: The Treatment of the Mentally Ill

Of interest was the treatment of the mentally ill, which during the period of the Ottoman Empire – which is a long period for which there is not much evidence – was done in asylum-type institutions and sometimes in churches and monasteries. Faith was thought to heal the mentally ill in a sense, while inhumane practices such as folk psychosurgery with red-hot irons were reported (Ploubidis, 1995).

The first asylum was established in Corfu, in 1838, by the British administration and was housed in the equestrian stables. The first doctors of the asylum were British, while the first Greek doctor, Christodoulos Tsirigotis, took over as director in 1874.

Already in Constantinople-during Ottoman Empire- there were several institutions that accepted the mentally ill. In the 16th century, we have the establishment of the hospital of Galata of Gemintzidon, which accepted the mentally ill.

In 1780 the hospital of Stadrodomiu, in 1839 of Heptapyrgio, in 1855 the La Paix asylum in Constantinople was founded by Catholic nuns, in 1748 the Greek hospital with an insane asylum department.

In Constantinople, the Ottomans founded two insane asylums in 1465, the Fatih Mosque Hospital and in 1527 the second one in the Suleiman Mosque Hospital. In 1583, Sultana Valide was founded and in 1850 the TopTahi Hospital, both of which received mental patients (Madianos 1994; Ploubidis, 1995).

At the end of the 19th century and the beginning of the 20th century, some forms of psychiatric treatment were available, as it was reported that hospitals in Chios, Smyrna, and Constantinople received mentally ill patients with various diagnoses such as primitive dementia, progressive general paralysis, and mania-melancholia (Ploubidis, 1995).

Opinions about mental illness in Greece were shaped according to the studies of Greek psychiatrists abroad. Some followed the German school and others the French school. Essentially, psychiatry was organized after 1930 and the founding of the Neurological and Psychiatric Society. The prevailing opinion was that psychiatric diseases derived from organic causes, and the therapeutic practices were similar, including bed rest, electric shock, drugs, cold and hot water, occupational therapy, and hydrotherapy.

Public psychiatric hospitals, such as the University Psychiatric Clinic Aeginetio founded at the start of the 20th century, were staffed by academic psychiatrists and treated patients using the well-known psychotherapeutic methods of the time. After the 1960s, psychosocial approaches such as drama groups, psychodrama, and group psychotherapy were also mentioned. More generally, however, the psychiatric world remained oriented toward the neurobiological and organic basis of mental disorders while promoting chemical treatments and expressing doubts about the findings of the new science of psychology and psychoanalysis. Over time, however, psychoanalysis was increasingly supported by a growing number of medical representatives. However, Greek society at that time – characterized by its low level of education, conservatism, orthodox Christian religion, and poor economic situation for large numbers of the population – generally maintained a negative attitude toward psychoanalysis and its emphasis on sexuality.

In addition to academic psychology, a network of mental health services was created in the context of social welfare in the early days of the 20^th^ century. Associations promoting mental health, institutions treating children’s psychosomatic health, and, since the mid-20th century, institutions for the blind and deaf, psycho–pedagogical centres, medical–pedagogical centres, psychotherapeutic clinics, vocational guidance centres, student perception centres, and psychological test institutes were established.

An important development for community mental health was the establishment in the 1950s, following a proposal by psychoanalyst Anna Potamianou, of the Centre for Mental Hygiene, which offered a series of counselling and therapeutic prevention and intervention services. A corresponding development was the Psychological Centre of Northern Greece in Thessaloniki in the 1960s, which aimed to address the broader mental health problems in Northern Greece. Its function was closely related to the scientific activity of Eftychia Nanakou, a doctor and psychologist who treated children with mental retardation. In the 1960s, the Athenian Centre for the Study of Man was founded by psychiatrist Georgios Vassiliou and psychologist Vaso Vassiliou as the first institute of systemic and family therapy, with methods based on family and group dynamics such as those taught at Loyola University of Chicago.

### After 1960 – The Consolidation of Clinical Psychology

During the period 1964–1997, the above processes were been completed, and clinical psychology was consolidated and developed into various fields. Hence, Greece saw the consolidation of the science of psychology in both academic instruction and scientific activity. At the Universities of Ioannina, Thessaloniki, and Athens, psychology courses were taught within the faculty of philosophy in the Departments of Philosophy, Pedagogy, and Psychology. Later the field of psychology became autonomous, and independent departments of psychology were created. Clinical psychology was taught alongside the other subjects of psychology during these years. Key representatives of applied psychology were Maria Nasiakou in Ioannina as a school psychologist, Mika Haritou Fatourou in Thessaloniki as a clinical psychologist, and Ioannis Paraskevopoulos and Anastasia Kalantzi-Azizi in Athens in psychometry and clinical psychology, respectively.

Applied Psychology, as we saw above, was taught as early as the 1930s in pedagogic departments, academies and teaching in the context of either general or special education. Educational and school psychology, developmental and evolutionary psychology, developmental psychopathology, assessment of intelligence and learning disabilities as well as therapeutic interventions are the main courses taught at this time, after 1960, in these schools. The 1970s saw the establishment of parenting schools through the work of psychologist Maria Khourdakis, which aimed to protect the mental health of both children and parents. These schools were based on the theories of family psychology, educational psychology, and school pedagogy. They had a significant impact on the public and 135 schools were operating nationwide by the 1980s.

Important in the consolidation and independence of the science of psychology in Greece was the establishment of the Association of Greek Psychologists in 1963 and the Panhellenic Psychological Association in 1997, which defended the rights of licensed professional psychologists. However, despite continuous efforts, psychologists in Greece have not succeeded in establishing a single association that constitutes a legal entity under public law. Additionally important is the presence of the Hellenic Psychological Society, a scientific association founded in 1990, that includes branches of clinical psychology and health psychology, among others. Apart from this organization, there is no separate association of clinical psychology in Greece.

Moreover, the connection between clinical psychology and psychotherapy lacks a clear framework in Greece. Psychotherapy is clearly a part of clinical psychology; for example, in the postgraduate programs of clinical psychology, psychotherapy is certainly a responsibility of the clinical psychologist. By contrast, many private institutes for the training of psychotherapists are not directly related to clinical psychology, as this discipline is taught in universities. It is imperative to institutionally clarify the professions of clinical psychologist and psychotherapist, a distinction that remains unclear.

Additionally, in the context of social welfare, various institutions have been established to accommodate children with various disabilities or those who have been removed from their families due to neglect or abuse. Children’s psychological support and therapeutic interventions, occupational therapy, psychosocial support, and special vocational training have been broadly applied in these structures.

At the same time, since the 1950s, various vocational orientation tests have been introduced in the psychological laboratories of Athens and Thessaloniki, while counselling for students has also been offered. From 2000 onwards, programs to remove social exclusion have been extended to various categories of people, such as prisoners, addicted people and disabled people, mainly with funding from the European Union.

#### Clinical Psychology, Psychiatry, and Psychiatric Reform

Clinical psychology in Greece remains a popular discipline among young psychologists. Its relationship with psychiatric medicine was partly conflictual due to the different backgrounds of the two sciences and the therapeutic practices that each proposes. However, the psychological training of doctors should be noted, with the introduction of psychology courses in medical schools in 1970 and the increasing participation of psychiatrists in psychotherapeutic training programs. At the same time, the contribution of clinical psychologists to psychiatric reform, oriented towards deinstitutionalization, community psychology, and social psychiatry, was important. The work of psychiatrists Kostas Stefanis, Panagiotis Sakellaropoulos, Petros Hartokollis, Stavroula Berati, Nikolaos Tzavaras, Charalampos Hierodiakonou, and Georgios Anastasopoulos and psychologists Anna Kokkevi, Alexandra Routsoni, R. Diakogianni, Maria Dolianiti, Ilias Fragos, and Thaleia Vergopoulou is important in this direction.

The operation of psychiatric hospitals and the structure of psychiatric reform from the 1980s onwards contributed to the promotion of clinical psychology in Greece. The same decade saw the establishment of the first addiction centres in Greece, KETHEA and 18 Ano, with the significant contributions of psychiatrists Phoebus Zafirides and Katerina Matsa, respectively. Psychiatrists, psychologists, social workers, and psychiatric nurses worked at these centres, comprising the interdisciplinary teams.

#### Clinical Child and Adolescent Psychology and Psychiatry

Child psychiatry organisations have been established since 1950 to treat the mental health of children and adolescents. An important activity for the psychosomatic health of children was developed at the child psychiatric clinic of the Agia Sophia Children’s Hospital, founded in 1978. This department provides services such as diagnostic consultation, short hospitalization and treatment, psychosocial support, and pre-professional training. The department trains doctors and psychologists and has prepared prevention programs. Psychiatrists and psychologists Gerasimos Stefanatos, Ioannis Tsiantis, Olga Maratou, and Anna Kokkevi pioneered this effort.

Also related to the practice of clinical psychology in Greece is the establishment of various organizations from 1960 onwards with the aim of providing psychotherapeutic services, the education and training of mental health professionals, and, sometimes, research. These groups followed various psychological approaches and practices and include the Hellenic Counselling Society; Hellenic Psychoanalytic Society; Hellenic Society for Dyslexia; Hellenic Society for Research and Behavior Therapy; Centre for Family Therapy; Alcoholics Anonymous; Centre for Individual Psychology; Centre for Psychotherapy and Gestalt Training; Hellenic Society for Community Therapists, Sociotherapy, and Psychodrama; and the Medical Sexology Institute. These societies have become widely associated with applied psychology, as many graduates of psychology departments have continued their education there to be trained in a psychotherapeutic approach, thus complementing the knowledge offered by academic study. The training in these organizations had a more practical orientation that psychology departments usually did not offer except at the postgraduate level.

## Academic Departments

Perhaps the most important event for the development of psychology in Greece and, by extension, clinical psychology was the establishment in the mid-1980s of independent university departments of psychology. The first Department of Psychology was founded in 1987 at the University of Crete. The contribution of psychologist Maria Khourdaki was important as, together with professors Ioannis Nestoros, Nikos Papadopoulos, and Grigoris Potamianos, she founded the new department. Following the example of the University of Crete, other psychology departments were established in Athens and Thessaloniki. All departments arose from philosophical schools with the exception of the one at Panteion University, which was rooted in the sociology department. The newly established Department of Psychology of Panteion University was staffed by professors of the Department of Sociology such as Stamos Papastamou, Foteini Tsalikoglou, and Aimilios Metaxopoulos.

In 1992, a Department (Program) of Psychology was established at the Kapodistrian University of Athens with professor George Paraskevopoulos and assistant professors Ilias Bezevegis, Nikolaos Giannitsas, and Anastasia Kalantzi-Azizi staffing this new department, which was part of the Department of Philosophy, Pedagogy, and Psychology. Postgraduate programs were not initially offered.

Since 1993, an autonomous Department of Psychology has been operating at the Aristotle University of Thessaloniki, which also emerged from the Department of Philosophy, Pedagogy, and Psychology. The department followed three directions – school–evolutionary, experimental–cognitive, and social–clinic – and was founded by professors Maria Maniou-Vakali, Anastasia Euclides, Dimitra Papadopoulou, Mika Charitou-Fatourou, Diomedes Markoulis, Dimitrios Natsopoulos, and Kostas Bayraktaris. Postgraduate studies have been offered almost from the beginning, while various scientific activities such as conferences, workshops, and seminars have been conducted.

The map of psychology departments in Greece was completed with the establishment of two new departments in 2019: the University of Western Macedonia, based in Florina, and the University of Ioannina, which also emerged from the older Department of Philosophy, Pedagogy, and Psychology.

## Instead of an Epilogue: Clinical Psychology in Greece Today

In Greece at the moment there are six psychology departments in public universities, four of which provide at least two years of postgraduate study in clinical psychology. Additionally, at least 10 affiliates of foreign universities have been established in which undergraduate studies in psychology are taught, and at least five offer postgraduate studies in clinical psychology. Private institutes providing training in various treatment approaches are numerous, and it is difficult to calculate their exact number.

The professional training of clinical psychologists, as can be deduced from the above, is provided at the postgraduate level mainly at public universities but also at private colleges. These programs have an academic structure and also provide extensive practical training.

As mentioned above, professional clinical psychology is not recognized officially and it is not institutionally protected. Hence, there are no financial benefits for people who use the services of clinical psychologists through Greece’s healthcare system. At the institutional level, there is the branch of clinical psychology in the Hellenic Psychological Society that validates this specialty.

Clinical psychologists in Greece work in both medical and psychiatric clinics, addiction treatment programs, and other therapeutic settings as well as on an outpatient basis or in private practice. In recent years, the positions of clinical psychologists in public institutions have been scarce, leading to more clinicians to be employed in private practices. The work of clinical psychologists in medical and psychiatric clinics includes diagnosis, therapy, consultation, crisis intervention, and supervision. In private practices, it involves mainly therapy and consultation.

Another important issue is the unclear distinction between clinical psychology and psychotherapy. Officially, neither is officially recognized as a profession in Greece. However, psychotherapy is undeniably part of the work of a clinical psychologist. Informally, psychotherapists are those who have completed training in a particular therapeutic approach, and their numbers are probably greater than clinical psychologists, but official figures are lacking. The public health system employs more clinical psychologists since academic qualifications, such as a master’s degree and a doctorate, are usually required.

Through the above-described developments, clinical psychology has established itself as a discipline in Greece. However, there are institutional gaps in care that must be addressed to secure and further develop the field of clinical psychology (see Kalantzi-Azizi & Karadimas, 2009). First and most importantly, the Greek legislation regarding the profession of psychologist includes no mention of registered specializations such as clinical, school, or cognitive psychology. Therefore, clinical psychology is taught at an academic level and practiced at a clinical level, but it does not have legal status as a specialization.

A second deficiency that undermines the field of clinical psychology is the fact that the licence to practice the profession of psychologist in Greece is issued directly after the completion of undergraduate studies, with only two months of practice. This is unprecedented, as a psychologist can typically undertake clinical work without having completed the necessary supervised clinical practice. The most basic requirement for a clinical psychologist should be supervised clinical practice for at least one year.

In conclusion, clinical psychology in Greece has a long history and has been served by notable scientists, initially from the field of medicine and later from psychology. However, the regulation by the state is needed to secure the field of clinical psychology in the present and to create the groundwork for its development in the future.

## Reporting Guidelines

The author has followed the EQUATOR Reporting Guidelines (https://www.equator-network.org) and has used the PRISMA guidelines (http://prisma-statement.org).
